# Point Mutations and Cytochrome P450 Can Contribute to Resistance to ACCase-Inhibiting Herbicides in Three *Phalaris* Species

**DOI:** 10.3390/plants10081703

**Published:** 2021-08-19

**Authors:** José G. Vázquez-García, Joel Torra, Candelario Palma-Bautista, Ricardo Alcántara-de la Cruz, Rafael De Prado

**Affiliations:** 1 Department of Agricultural Chemistry, Edaphology and Microbiology, University of Córdoba, 14014 Córdoba, Spain; z82pabac@uco.es; 2 Department d’Hortofruticultura, Botànica i Jardineria, Agrotecnio, Universitat de Lleida, 25198 Lleida, Spain; joel.torra@udl.cat; 3 Centro de Ciências da Natureza, Campus Lagoa do Sino, Universidade Federal de São Carlos, Buri 18290-000, Brazil; ricardo.cruz@ufscar.br

**Keywords:** herbicide resistance, resistance mechanisms, NTSR mechanisms, TSR mechanisms, metabolism

## Abstract

Species of *Phalaris* have historically been controlled by acetyl-coenzyme A carboxylase (ACCase)-inhibiting herbicides; however, overreliance on herbicides with this mechanism of action has resulted in the selection of resistant biotypes. The resistance to ACCase-inhibiting herbicides was characterized in *Phalaris brachystachys*, *Phalaris minor,* and *Phalaris paradoxa* samples collected from winter wheat fields in northern Iran. Three resistant (R) biotypes, one of each *Phalaris* species, presented high cross-resistance levels to diclofop-methyl, cycloxydim, and pinoxaden, which belong to the chemical families of aryloxyphenoxypropionates (FOPs), cyclohexanediones (DIMs), and phenylpyrazolines (DENs), respectively. The metabolism of ^14^C-diclofop-methyl contributed to the resistance of the *P. brachystachys* R biotype, while no evidence of herbicide metabolism was found in *P. minor* or *P. paradoxa*. ACCase in vitro assays showed that the target sites were very sensitive to FOP, DIM, and DEN herbicides in the S biotypes of the three species, while the R *Phalaris* spp. biotypes presented different levels of resistance to these herbicides. ACCase gene sequencing confirmed that cross-resistance in *Phalaris* species was conferred by specific point mutations. Resistance in the *P. brachystachys* R biotype was due to target site and non-target-site resistance mechanisms, while in *P. minor* and *P. paradoxa*, only an altered target site was found.

## 1. Introduction

The genus *Phalaris* L. has a complicated taxonomic history. This genus comprises 22 species of annual and perennial grasses found in open habitats of temperate regions around the world, affecting cereal, pasture fodder, and vegetable crops [1]. *Phalaris* spp. are among the most frequent annual winter weeds in Iran, and they are represented mainly by *Phalaris minor* Retz., *Phalaris paradoxa* L., and *Phalaris brachystachys* Link. [2]. These species are distributed in various regions of the country, invading mainly wheat fields and other arable crops [3,4]. In Iran, wheat is the most important crop, while weeds, mainly *Avena* spp., *Lolium* spp., and *Phalaris* spp. grasses, can reduce the annual yield by ~23% [5]. In addition, *Phalaris* spp. are highly competitive plants with high seed production [6,7,8]; therefore, managing these grasses is essential to avoid compromising crop yields.

Acetyl-coenzyme A carboxylase (ACCase)-inhibiting herbicides (WSSA/HRAC group 1/A) are graminicides widely used to control grass weeds, mainly in cereal fields [9]. Their post-emergence control of grass weeds in a wide variety of field crops accounts for their intensive use since their introduction [10]. These graminicides inhibit the plastid form of ACCase by blocking fatty acid biosynthesis, disrupting cell membrane integrity, and consequently causing metabolite leakage and rapid plant death [11]. Repeated applications of ACCase inhibitors, sometimes two or three times per crop season, have led to the selection of resistant plants of several grass weed species worldwide [12]. Herbicide resistance is an adaptive evolutionary process, and its dynamics and impacts depend on various factors, such as genetic diversity, weed biology, herbicidal and operational components, and other environmental factors [10]. The resistance of grass weeds to ACCase inhibitors is steadily increasing worldwide [4]. After the introduction of the chemical families of aryloxyphenoxypropionates (FOPs), cyclohexanediones (DIMs), and phenylpyrazolines (DENs), resistance to ACCase inhibitors was reported in *Lolium rigidum* and *Alopecurus myosuroides*, and within a few years resistance had spread to other grass weeds [4]. ACCase inhibitors have been the only postemergence herbicides available for selective control of grass weeds in Iran [13]. The most troublesome annual grass weeds found in Iranian wheat fields are *L. rigidum*, *Phalaris* spp. and *Avena sterilis* L. During the last decade, these species have evolved several ACCase-resistant populations showing differential patterns of cross-resistance [11,14,15].

Resistance to ACCase inhibitors in grass weeds may be due to two known mechanisms: (1) alterations of the gene encoding the herbicide target site (target-site-based resistance: TSR) [11,16]; (2) a reduction in the amount of active herbicide molecules reaching their target due to enhanced metabolism and reduced foliar penetration (non-target-site resistance—NTSR) [17,18]. Regarding TSR, different amino acid substitutions at key positions in the carboxyl transferase (CT) domain have been reported at amino acid positions 1781, 1999, 2027, 2041, 2078, 2088, and 2096 in different species [5,16,19,20]; however, there is only one documented case of ACCase overexpression [21].

Both TSR and NTSR mechanisms have been reported in *Phalaris* spp. populations with resistance to ACCase inhibitors, including in Iran and neighboring countries of the Middle East [22,23,24]. Because ACCase inhibitors have been applied for at least ten years in the northern regions of Iran, populations of *Phalaris* spp. may have developed increased resistance to ACCase-inhibiting herbicides. In this study, we describe and compare the biochemical and molecular aspects involved in the resistance to ACCase-inhibiting herbicides among three *Phalaris* species (*P. minor*, *P. paradoxa,* and *P. brachystachys*) collected in winter wheat fields in Iran in 2018.

## 2. Results

### 2.1. Dose Response Assay

Dry weight values gradually decreased in all *Phalaris* spp. populations as the doses of herbicides increased; however, the dry weight reductions differed between species depending on the herbicide. The S populations of the three *Phalaris* spp. were effectively controlled with lower doses than the field doses of diclofop-methyl (DM), cycloxydim, and pinoxaden (900, 250, and 40 g ai ha^−1^, respectively). The GR_50_ values of the S populations ranged from 129.56 to 244.90 g ai ha^−1^ for DM, while for cycloxydim and pinoxaden, these values were below 10.5 g ai ha^−1^ (Table 1). The *P. brachystachys* R populations showed resistance to DM (RF = 10.31), although the RF values for cycloxydim and pinoxaden were 4.48 and 5.38, respectively. In addition, the GR_50_ values were much lower than the field doses of DIM and very similar to the field doses of DEN herbicides. R populations of *P. minor* and *P. paradoxa* showed cross-resistance to DM (RF = 7.48 and 11.87, respectively), cycloxydim (RF = 19.65 and 24.05, respectively), and pinoxaden (RF = 6.81 and 17.12, respectively).

### 2.2. ^14^C-DM Metabolism

When plants were not incubated with ABT, the ^14^C-DM metabolism patterns were similar between the *P. minor* and *P. paradoxa* R and S populations but not in *P. brachystachys* populations. For the latter populations, the transformation rate of DM acid (toxic form) into polar conjugates of ^14^C-DM (non-toxic metabolites) was ~3 times greater in the R biotype than in its S counterpart (Table 2). Pretreatment with ABT solution severely decreased this metabolic rate in the *P. brachystachys* R population, and the concentrations of DM acid and polar conjugates reached concentrations similar to those observed in the S population and in the other R and S populations of *P. minor* and *P. paradoxa*. In these populations, the percentages of DM, DM acid, and D-conjugates ranged from 29 to 35%, from 55 to 67%, and from 11 to 16%, respectively.

### 2.3. ACCase Enzyme Activity Assay

**Figure 1:**
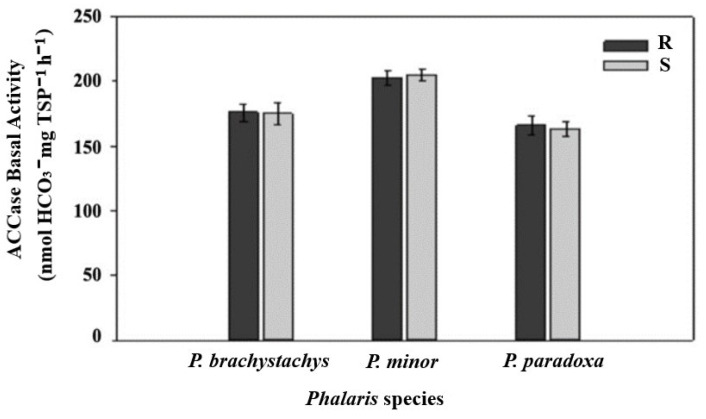
Basal ACCase activity (absence of herbicides) in R and S biotypes of *Phalaris* species expressed as nmol HCO_3_ per mg of total soluble protein (TSP) per hour.

R and S plants presented similar ACCase basal activity profiles in the absence of herbicides within *Phalaris* spp. (Figure 1). ACCase assays showed that the target site of the S populations was very sensitive to the three herbicides tested, and in all cases I_50_ was ≤1 µL in the R populations of *Phalaris* spp. (Table 3). ACCase insensitivity was variable, showing I_50_ values that ranged from 2.4 to 14.43 µL of herbicide. The RF values for R biotypes ranged from 12.99 to 20.78 for DM, from 5.16 to 10.91 for cycloxydim, and from 13.71 to 19.36 for pinoxaden.

### 2.4. Molecular Analysis

**Figure 2:**
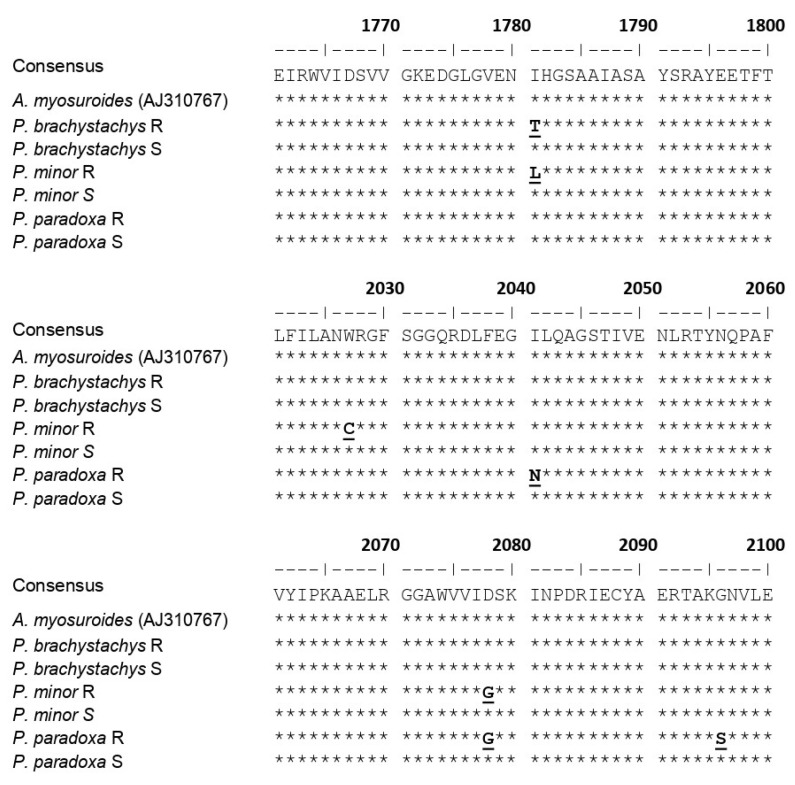
Amino acid substitutions in the A and B regions of the carboxyl transferase (CT) domain of the homomeric chloroplast acetyl CoA carboxylase (ACCase) gene in susceptible (S) and resistant (R) *Phalaris* biotypes.

CT-domain sequencing of the *ACCase* gene revealed the occurrence of several amino acid substitutions in the R biotypes of *Phalaris* spp., all of them associated with resistance to ACCase-inhibiting herbicides at the target site level (Figure 2). The amino acid substitutions found in the R biotypes were Ile-1781-Leu (L) or Thr (T), Trp-2027-Cys (C), Ile-2041-Asn (N), Asp-2078-Gly (G), and Gly-2096-Ser (S). In *P. brachystachys*, only the substitution of Thr for Ile at position 1781 was found. *P. minor* showed Ile-1781-Leu, Trp-2027-Cys, and Asp-2078-Gly substitutions, while *P. paradoxa* showed the substitutions of Ile-2041-Asn, Asp-2078-Gly, and Gly-2096-Ser.

## 3. Discussion

Since the first study to detect weed biotypes resistant to herbicides in Iran in 1997, several species, including *Avena sterilis*, *L. rigidium*, *P. minor,* and *P. paradoxa*, have shown resistance to ACCase inhibitors in this country [25]. These reports of resistant weeds were found in winter cereals in Iran, mostly wheat (*Triticum aestivum* L.) and barley (*Hordeum vulgare L*.) [26]. Herbicide-resistant biotypes of *P. minor* are widely distributed around the world, while resistant biotypes of *P. paradoxa* and *P. brachystachys* are less frequent; however, these three species have been reported as having cross-resistance to all ACCase-inhibitor herbicide chemical families [4]. It is probable that the few grass herbicide options for wheat in Iran and the lack of crop rotation aided in the selection of target site mutations in *Phalaris* species and their consequent resistance to ACCase inhibitors [13].

In this study, dose–response experiments confirmed that the three *Phalaris* spp. assayed at the whole-plant level were resistant to DM, cycloxydim, and pinoxaden. These results are in agreement with those of other studies that reported similar resistance patterns to the three groups of ACCase-inhibiting herbicides, such as those in *P. minor* [16,23], *P. paradoxa* [22,26,27], and *P. brachystachys* [24].

Enhanced metabolism, regulated by detoxification enzymes Cyt-P450, glutathione-S-transferase (GST), and Glycosyl-transferase (GT), which are able to metabolize herbicides into non-toxic metabolites, may confer resistance to herbicides [28,29,30,31,32]; however, we found no evidence that enhanced metabolism conferred resistance in *P. minor* and *P. paradoxa*, in agreement with the results found by Cruz-Hipolito et al. [16,27], while for *P. brachystachys*, this NTSR mechanism, mediated by the enzyme complex of Cyt-P450 monooxygenases, was responsible for resistance to DM. This statement is based on the response observed in *P. brachystachys* R plants incubated with ABT, a potent inhibitor of Cyt-P450, which after incubation showed ^14^C-DM metabolism patterns similar to those of S plants. In general, in susceptible plants, DM is de-esterified (activated) by an esterase enzyme into diclofop acid, a compound more toxic than DM [17,33]. On the other hand, in R plants, DM is metabolized into non-toxic compounds that are more polar, such as sugar ester conjugates of diclofop acid and sugar conjugates of hydroxyl-diclofop, by Cyt-P450 [24]. DM metabolization into sugar conjugates of hydroxyl-diclofop seems to be the main detoxification route of this herbicide in *P. brachystachys*. Enhanced herbicide metabolism is an NTSR mechanism documented in several grass weeds around the world, such as *L. rigidum* [17,34], *Alopecurus myosuroides* [35], and *Echinochloa phyllopogon* [36], and in recent years this mechanism has gained more attention because it is becoming an increasingly common resistance mechanism, although comprehension of this mechanism is still limited [31]. Cyt-P450-regulated herbicide metabolism can confer not only cross-resistance but also multiple resistance to modes of action of herbicides that are never used [37]. Although multiple resistance was not evaluated in the R biotype of *P. brachystachys*, its management may require more diversification than simply rotating the mode of action.

The ACCase enzyme activity results showed clear cross-resistance to the three ACCase-inhibiting herbicides in the *Phalaris* spp. R biotypes at the target site level. The resistance was high to pinoxaden and diclofop methyl and moderate to cycloxydim. According to these results, a less sensitive form of ACCase was responsible for cross-resistance to ACCase-inhibiting herbicides in R populations; however, each R population of *Phalaris* spp. showed a different susceptibility level for each herbicide. The level of insensitivity of the target enzyme to ACCase-inhibiting herbicides depends on amino acid substitutions at key positions that modify the binding of the herbicide [10,18,33]; therefore, the mutations governing herbicide resistance may differ among *Phalaris* spp.

Target site resistance is essentially caused by amino acid changes in the CT domain, which impact the effective binding of ACCase-inhibiting herbicides [10]. Substitutions in seven locations (Ile-1781, Trp-1999, Trp-2027, Ile-2041, Asp-2078, Cys-2088, and Gly-2096) in the ACCase gene have been described in grass weeds as conferring resistance to ACCase inhibitors [18]. In this study, six different substitutions were found at five key amino acid positions (Ile-1781-Leu/Thr, Trp-2027-Cys, Ile-2041-Asn, Asp-2078-Gly, and Gly-2096-Ser) in the ACCase genes of the R biotypes of the *Phalaris* spp. The substitutions found at key position 1781 occurred in *P. minor* (Ile by Leu) and *P. brachystachys* (Ile replaced by Thr). Mutation Ile-1781-Leu has been reported in R populations of *P. minor* and other grass species resistant to ACCase herbicides [16], conferring cross-resistance to the three chemical families (FOPs, DIMs, and DENs). The Ile-1781-Thr mutation has been found in *P. brachystachys* and *A. myosuroides* [18,24] and was suggested to confer resistance mainly to FOP and DEN herbicides. In the same form, Trp-2027-to-Cys substitution endows resistance to ACCase inhibitors [23], mainly to FOPs [18,19,38,39], although also confers resistance to DENs [20]. This mutation was found in *P. minor* but not *P. paradoxa* or *P. brachystachys*. In addition to the mutation at the Ile-1781 position, the Asp-2078-Gly mutation was found to confer resistance to the three families of ACCase inhibitors [22,38]; therefore, this mutation explains the cross-resistance to DM, cycloxydim, and pinoxaden in our *P. minor* and *P. paradoxa* R biotypes. On the other hand, the Ile-2041-Asn and Gly-2096-Ser mutations were found only in *P. paradoxa*. These mutations have been previously reported in *P. paradoxa* from Israel [22] and Mexico [27]. The first mutation was related to resistance only to FOPs [20], while the second was associated with cross-resistance to FOPs, DIMs, and DENs [27].

## 4. Materials and Methods

### 4.1. Plant Materials

Seeds of resistant (R) populations of *Phalaris* spp. were collected from winter wheat fields from Golestan Province in Iran (Figure S1, Table S1, Supplementary Materials). The R *P. brachystachys* and *P. minor* plants survived field applications of diclofop-methyl (DM), cycloxydim, and pinoxaden herbicides, while *P. paradoxa* presented medium resistance levels to these herbicides, with low frequencies of R individuals; therefore, generation 0 (G0) plants from R *P. paradoxa* population were sown directly into trays (40 × 60 × 15 cm^3^) containing a mixture of sand and peat (2:1, *v/v*) and placed in a greenhouse at 28/18 °C day/night under a 16 h photoperiod with 850 µmol m^−2^ s^−1^ photon flux density and 80% relative humidity. At the four-leaf stage, plants were treated with DM at 300 g ai ha^−1^, one hour later with cycloxydim at 100 g ai ha^−1^, and finally one hour later with pinoxaden at 30 g ai ha^−1^, using a laboratory spray chamber equipped with a flat fan nozzle (TeeJet 8002 EVS) with a total output volume of 250 L ha^−1^ water at a pressure of 200 kPa. Four weeks after herbicide treatment, plant survival of the resistant accessions was estimated, and seeds produced from surviving plants were collected and stored in paper bags for subsequent recurrent selection trials. Five months later, G1 seeds of *P. paradoxa* were treated as above, although in this case with field doses of 900, 250, and 40 g ai ha^−1^ of DM, cycloxydim, and pinoxaden, respectively. A similar approach was followed to produce G2 and G3. In January 2020, dose–response assays were run to determine the multiple resistance levels in these three *Phalaris* species. Seeds of susceptible (S) populations were harvested within the same region in sites where herbicides had never been applied. Mature R and S seeds of *P. brachystachys* and *P. minor* and F3 *P. paradoxa* populations were germinated in Petri dishes (15 cm diameter) with filter paper moistened with distilled water. The Petri dishes were placed in a growth chamber at 28/18 °C (day/night) with a photoperiod of 16 h, 850 μmol m^−2^ s^−1^ photosynthetic photon flux, and 80% relative humidity. Seedlings from each population were transplanted individually into plastic pots (448 cm^3^) containing sand/peat at a 1:2 (*v/v*) ratio, then they were then placed in a greenhouse at 28/18 °C (day/night) with the same photoperiod.

### 4.2. Dose–Response Assays

Plants of the R and S biotypes of *Phalaris* spp. were treated at the BBCH 13–14 stage [40] with different doses of DM, cycloxydim, and pinoxaden (Table 4). Ten plants of each biotype were treated per herbicide dose in a laboratory spray chamber equipped with a Tee Jet 8002E-VS flat-fan nozzle at a pressure of 200 kPa calibrated to deliver 250 L ha^−1^ of herbicide solution. Twenty-one days after treatment (DAT), the plants were cut and oven-dried to constant mass at 70 °C, then the dry weight of each plant was recorded. Dry weight data were expressed as percentages relative to the untreated controls. The herbicide rates required to reduce the dry weight by 50% (GR_50_) were determined for each biotype and the resistance factors (RFs) were determined for each herbicide within each species as GR_50_(R)/GR_50_(S). Dose–response assays were repeated twice.

### 4.3. ^14^C-DM Metabolism

^14^C-DM metabolism was studied following the methodologies described by Cruz-Hipolito et al. [27] and De Prado et al. [17]. A labeled herbicide emulsion was prepared by mixing commercially formulated DM plus ^14^C-DM (specific activity, 95.5 kBq µmol^−1^ provided by Bayer CropScience, Leverkusen, Germany) (Table 2). The ^14^C-DM emulsion had a specific activity of 5000 Bq µL^−1^ and was applied to the adaxial surface of the second leaves (10 droplets of 0.5 μL each) of *Phalaris* spp. plants (BBCH 13–14 stage) using a microapplicator (mod. PB600-1 Hamilton, Reno, NV, USA). Sampling of plants was carried out 48 h after treatment (HAT), starting by washing the non-absorbed ^14^C-herbicide from the treated leaves with 1.5 mL of acetone. An aliquot of leaf wash solution was assayed for radioactivity and the remaining solution was stored (−20 °C) until analysis. Then, the shoots of each plant were ground in liquid nitrogen in a cold mortar. ^14^C-DM and its possible metabolites were extracted from the fine powder with 4 mL of methanol (80% methanol, 4 °C). The homogenate was centrifuged (20,000× *g* for 20 min). The resulting pellet was subjected to two new extractions with methanol (80%) to recover as much ^14^C as possible. The pellets were oven-dried and combusted in a biological oxidizer (Packard 307, Packard Instruments, Meriden, CT, USA). Supernatants were combined and evaporated to dryness (40 °C) under a stream of N_2_ (10 kPa), then samples were redissolved in 500 µL of methanol (80%). DM and its metabolites in the supernatant were identified using thin-layer chromatography on 20 cm × 20 cm × 250 μm silica gel plates (Merck, Darmstadt, Germany; silica gel 60). A toluene–ethanol–acetic acid mixture (150/7/7; *v/v/v*) was used as the mobile phase. Radioactive zones were detected using a radio chromatogram scanner (Berthold LB 2821). Their chemical identity was identified by comparing RF values to those of standards (DM, 0.70; diclofop acid, 0.44; hydroxy-diclofop, 0.34; polar conjugates, 0.00). The experiment was performed twice with three replicates.

In a second experiment, three plants (BBCH 12–13 stage) per *Phalaris* spp. biotype were collected from the pots and the roots were washed with distilled water. Subsequently, plants were placed into 50 mL containers filled with a constantly aerated nutrient solution containing 7.5 mg L^−1^ 1-aminobenzotriazole (ABT), a potent inhibitor of the enzyme necessary for the metabolism of DM to non-toxic forms in plants, i.e., Cyt-P450 monooxygenases [27]. After 1 week of incubation with ABT, the second leaf of each plant was treated with ^14^C-DM 48 HAT and the methodology described above was followed again.

### 4.4. ACCase Enzyme Activity Assay

Young and fully expanded leaves (6 g fresh weight) of *Phalaris* spp. R and S populations were harvested from plants at the BBCH 13–14 stage. Leaf tissue samples were ground in liquid N_2_ in a mortar and added to extraction buffer (24 mL) composed of 0.1 M *N*-2-hydroxyethylpiperazine-*N*′-2-ethanesulfonic acid-KOH at pH 7.5, 0.5 M glycerol, 2 mM EDTA, and 0.32 mM PMSF. The homogenate was mixed for 3 min with a magnetic stirrer and filtered sequentially through four layers of cheesecloth. The crude extract was centrifuged (24,000× *g*, 30 min, 4 °C). The supernatant was fractionated with (NH_4_)_2_SO_4_ and centrifuged (12,000× *g*, 10 min, 4 °C). Material precipitating between 35% and 45% (NH_4_)_2_SO_4_ saturation was resuspended in 1 mL of S400 buffer (0.1 M Tricine–KOH at pH 8.3, 0.5 M glycerol, 0.05 M KCl, 2 mM EDTA, and 0.5 mM DTT). The clarified supernatant was applied to a desalting column previously equilibrated with S400 buffer. ACCase enzyme was eluted from the column in 2 mL of S400 buffer.

The enzyme activity was assayed by measuring the ATP-dependent incorporation of NaH [^14^C]O_3_ into an acid-stable [^14^C]-product. The reaction product had previously been shown to be [^14^C] malonyl-CoA [41]. Assays were conducted in (7 mL) scintillation vials containing 0.1 M tricine-KOH (pH 8.3), 0.5 M glycerol, 0.05 M KCl, 2 mM EDTA, 0.5 mM DTT, 1.5 mM ATP, 5 mM MgCl_2_, 15 mM NaH [^14^C]O_3_ (1.22 MBq μmol^−1^), 50 μL of the enzyme fraction, 5 mM acetyl-CoA, and different concentrations of DM acid for a final volume of 0.2 mL. Activity was assayed for 5 min at 34 °C and the reaction was stopped by adding HCl (30 μL at 4 N). A piece of filter paper was added to the reaction vial and the sample was dried (40 °C) under a stream of air. After the sample was dried, an ethanol–water mixture (1:1, *v/v*, 0.5 mL) was added to the vial. This was followed by the addition of a scintillation cocktail (5 mL). Radioactivity was determined by liquid scintillation spectrometry (LSS) on a Beckman LS 6000 TA. Background radioactivity values, measured as acid-stable counts (dpm) in the absence of acetyl-CoA, were subtracted from each treatment (16, 17, 24, 27). One unit of ACCase activity was defined as 1 μmol malonyl-CoA formed per min. Herbicide concentrations resulting in 50% inhibition of enzyme activity (I_50_ values) were estimated for each herbicide using the obtained concentration response curves. The experiment was repeated twice with three replicates. Data were pooled and a non-linear regression model (Equation (1)) was fitted to the data.

### 4.5. Molecular Analysis

Samples (~100 mg) of young foliar tissue were taken from 20 plants of the *Phalaris* spp. R and S biotypes. Then, the plants were treated with doses of DM, cycloxydim, and pinoxaden (900, 250, and 40 g ai ha^−1^, respectively), as described in the dose–response assays. S plants died 21 DAT and surviving R-biotype plants were used for molecular analysis. DNA was extracted using the Speedtools Plant DNA Extraction Kit (Biotools B&M Labs S.A., Spain) following the manufacturer’s instructions, and the DNA amount was quantified with a NanoDrop spectrophotometer (ThermoFisher, NanoDrop Products, Wilmington, DE, USA). The DNA sample was diluted to a final concentration of 10 ng/μL and was immediately used for polymerase chain reaction (PCR) or stored at −20 °C until use. Primers were designed to amplify regions in the CT domain known to be involved in the sensitivity to ACCase herbicides using Primer Premier 5.0 software (Premier Biosoft International, Palo Alto, CA, USA). Two sets of primers covering all seven known mutation sites in regions A (1781) and B (1999, 2027, 2041, 2078, 2088, and 2096) were designed based on the sequences of chloroplastic ACCase from *A. myosuroides* (AJ310767). Ten individual plants of each biotype were sequenced following the methods described by Golmohammadzadeh et al. [24].

### 4.6. Statistical Analysis

Dose–response and enzyme activity data were pooled and fitted to a non-linear regression analysis using a three-log-logistic model (Equation (1)), with the lower asymptote (c) fixed at 0 for GR_50_ and I_50_.

(1) $$Y=c+\;\frac{d-c}{1+\;\;\exp\left(b\left(\log\left(x\right)-\log\left(e\right)\right)\right)}$$

In the logistic model, *d* and *c* are the coefficients corresponding to the upper and lower (fixed at 0) asymptotes, respectively; *b* is the slope of the curve; *e* is the herbicide rate (or concentrations) at the point of inflection halfway between the upper and lower asymptotes; *x* (independent variable) is the herbicide dose (or concentration). Non-linear regression analyses were carried out in R software using the “drc” statistical package [42]. Data on DM metabolism and basal activity were subjected to analysis of variance (ANOVA). The percentage data were previously transformed (arcsine of the square root) to comply with the model assumptions of normally distributed errors and homogeneity of variances. The assumptions of the model were graphically inspected. Values of *p* < 0.05 were considered statistically significant and mean comparisons were made using Tukey’s HSD test with a probability of 5%. ANOVA was conducted using Statistix software version 10.0 (Analytical Software, Tallahassee, FL, USA).

## 5. Conclusions

This study characterized cross-resistance patterns to ACCase-inhibiting herbicides in populations of *Phalaris* spp. collected in wheat fields in Iran. It should be noted that the metabolic resistance governed by Cyt-P450 found in *P. brachystachys* is a cause for concern because it may provide widespread resistance to other modes of action and compromise crop productivity; therefore, its management may require more diversification than simply rotating the herbicide’s mode of action. On the other hand, choosing a suitable herbicide to control the biotypes of *Phalaris* spp. with cross-resistance patterns to ACCase inhibitors is risky due to the fact that a single amino acid substitution may lead to different levels of resistance. In addition, the continued use of ACCase inhibitors may facilitate the appearance of new Cyt-P450 genes in *P. minor* and *P. paradoxa*, as reported here for *P. brachystachys*, or new ACCase mutations able to confer higher levels of resistance to these herbicides. These results demonstrated the participation of TSR and NTSR mechanisms in the resistance to ACCase-inhibiting herbicides in the *P. brachystachys* R population, while only the TSR mechanism was involved in the resistant populations of *P. minor* and *P. paradoxa*.

## Figures and Tables

**Table 1:** Estimated parameters of the log-logistic equation used to calculate the herbicide doses required for 50% reduction of the dry weights (GR_50_) of R and S biotypes of *Phalaris* species.

Herbicides	Species	Biotype	d	b	GR_50_ (g ai ha^−1^)	RF ^a^
**Diclofop-methyl** **(FOP)**	*P. brachystachys*	R	99.04 ± 2.09	3.66 ± 0.79	1336.45 ± 109.29	10.31 ± 0.68
		S	98.13 ± 2.10	1.21 ± 0.57	129.56 ± 7.52	-
	*P. minor*	R	95.21 ± 3.09	3.85 ± 0.77	1832.34 ± 103.98	7.48 ± 0.73
		S	96.18 ± 3.81	2.88 ± 0.75	244.90 ± 19.71	-
	*P. paradoxa*	R	95.69 ± 11.15	2.74 ± 0.93	2831.61 ± 129.18	11.87 ± 1.57
		S	92.17 ± 7.43	1.63 ± 0.45	238.62 ± 31.87	
**Cyclocydim (DIM)**	*P. brachystachys*	R	99.94 ± 2.14	1.73 ± 0.49	39.61 ± 2.12	4.48 ± 0.25
		S	95.48 ± 1.10	1.42 ± 0.57	8.84 ± 0.95	-
	*P. minor*	R	97.3 ± 5.55	1.77 ± 0.30	198.44 ± 25.13	19.65 ± 1.89
		S	98.98 ± 5.73	1.33 ± 0.19	10.10 ± 1.63	
	*P. paradoxa*	R	96.19 ± 4.44	2.09 ± 0.37	236.85 ± 22.67	24.05 ± 2.74
		S	99.44 ± 4.67	1.47 ± 0.18	9.85 ± 1.21	-
**Pinoxaden** **(DEN)**	*P. brachystachys*	R	98.50 ± 4.74	0.69 ± 0.11	41.02 ± 3.89	5.38 ± 0.74
		S	99.71 ± 2.57	1.23 ± 0.46	7.63 ± 0.41	-
	*P. minor*	R	97.21 ± 5.88	1.48 ± 0.25	56.87 ± 9.25	6.81 ± 1.58
		S	96.87 ± 9.25	1.34 ± 0.21	8.35 ± 1.38	-
	*P. paradoxa*	R	96.81 ± 4.56	1.72 ± 0.37	116.95 ± 17.28	17.12 ± 1.62
		S	97.37 ± 6.04	1.63 ± 0.28	6.83 ± 1.03	-

*d* is the coefficient corresponding to the upper limits of the asymptotes and *b* is the slope of the curve; ^a^ Resistance factor (RF = GR_50_ resistant biotype (R)/GR50 susceptible biotype (S)). FOP, aryloxyphenoxypropionates; DIM, cyclohexanediones; and DEN, phenylpyrazolines.

**Table 2:** ^14^C-Diclofop-methyl (DM) percentage and metabolites retrieved from shoots after application to leaves in resistant (R) and susceptible (S) biotypes of *Phalaris* species 48 h after treatment (HAT).

*Phalaris* Species	% Extracted Radioactivity					
	DM		D-Acid		D-Conjugates	
	R	S	R	S	R	S
***P. brachystachys*** **†**	12.53 ± 2.13 b	26.72 ± 2.48 a	22.43 ± 1.83 b	67.50 ± 3.17 a	65.12 ± 3.42 a	16.83 ± 1.31 a
***P. brachystachys*** **‡**	30.41 ± 4.24 a	28.71 ± 3.52 a	59.87 ± 1.05 a	58.65 ± 2.19 a	16.80 ± 0.81 b	15.61 ± 0.54 a
***P. minor*** **†**	33.33 ± 1.45 b	35.18 ± 1.73 a	55.33 ± 4.33 a	56.66 ± 2.60 a	11.33 ± 2.90 b	12.37 ± 2.40 a
***P. minor*** **‡**	34.33 ± 2.02 b	33.33 ± 1.45 a	62.04 ± 2.30 a	62.13 ± 1.52 a	12.66 ± 1.45 b	11.33 ± 0.66 a
***P. paradoxa*** **†**	29.11 ± 0.47 b	32.33 ± 2.22 a	57.33 ± 2.37 a	56.33 ± 1.18 a	16.43 ± 1.88 b	14.56 ± 1.08 a
***P. paradoxa*** **‡**	29.66 ± 1.18 b	30.66 ± 1.18 a	67.66 ± 1.65 a	66.66 ± 0.72 a	13.66 ± 0.54 b	12.39 ± 0.82 a

1-Aminobenzotriazole (ABT) was applied via root at 7.5 mg L^−1^ one week before DM application. **†** Without ABT and **‡** with ABT. Different letters per column refer to treatments that are significantly different based on the Tukey test at the 95% probability. Mean values ± standard errors of the mean are shown (*n* = 6).

**Table 3:** Estimated parameters of the log-logistic equation used to calculate the enzyme activity levels (I_50_) of R and S biotypes of *Phalaris* species.

Herbicide	Species	Biotype	d	b	I_50_ (µM)	RF ^a^
**Diclofop-methyl** **(FOP)**	*P. brachystachys*	R	99.64 ± 0.83	0.67 ± 0.03	6.65 ± 0.78	20.78
		S	101.99 ± 0.23	0.72 ± 0.02	0.32 ± 0.05	-
	*P. minor*	R	94.90 ± 3.68	0.56 ± 0.09	11.04 ± 4.57	12.99
		S	96.49 ± 4.56	0.49 ± 0.07	0.85 ± 0.03	-
	*P. paradoxa*	R	93.98 ± 3.46	0.55 ± 0.08	9.73 ± 3.80	16.37
		S	97.91 ± 4.26	0.48 ± 0.06	0.59 ± 0.23	-
**Cyclocydim** **(DIM)**	*P. brachystachys*	R	103.11 ± 1.40	0.52 ± 0.03	2.40 ± 0.34	10.91
		S	100.53 ± 1.58	0.69 ± 0.04	0.22 ± 0.02	-
	*P. minor*	R	96.98 ± 2.90	1.76 ± 0.43	3.46 ± 1.87	5.16
		S	97.87 ± 2.81	0.98 ± 0.57	0.67 ± 0.91	-
	*P. paradoxa*	R	98.76 ± 0.76	0.87 ± 0.32	2.86 ± 0.98	6.21
		S	96.67 ± 0.67	1.11 ± 0.12	0.46 ± 0.06	-
**Pinoxaden** **(DEN)**	*P. brachystachys*	R	100.60 ± 1.40	0.90 ± 0.09	14.43 ± 1.90	18.50
		S	102.93 ± 2.22	0.62 ± 0.06	0.78 ± 0.01	
	*P. minor*	R	97.76 ± 2.76	0.78 ± 0.98	8.09 ± 2.80	13.71
		S	96.56 ± 1.98	0.87 ± 0.19	0.59 ± 0.07	-
	*P. paradoxa*	R	97.87 ± 2.98	0.76 ± 0.09	12.78 ± 2.87	19.36
		S	95.56 ± 3.98	1.09 ± 0.09	0.66 ± 0.09	-

*d* is the coefficient corresponding to the upper limits of the asymptotes and *b* is the slope of the curve; ^a^ Resistance factor (RF = GR_50_ resistant biotype (R)/GR50 susceptible biotype (S)). FOP, aryloxyphenoxypropionates; DIM, cyclohexanediones; and DEN, phenylpyrazolines.

**Table 4:** Herbicide treatments for dose–response assays in *Phalaris* species.

Herbicides	Rate (g a.i. ha^−1^)	
	Biotype S	Biotype R
Diclofop-methyl ^a^	0, 45, 90, 180, 360, 720, 1080	0, 1000, 1500, 2000, 3000, 3500, 4000,
Cycloxydim ^b^	0, 5, 10, 20, 40, 60, 100, 200	0, 50, 100, 200, 300, 400, 800,1200
Pinoxaden ^c^	0, 4, 8, 16, 32, 64, 128, 256	0, 25, 50, 100, 200, 400, 600, 800

Trademark: ^a^ Iloxan (Bayer). ^b^ Focus Ultra 10% (BASF). ^c^ Axial (Syngenta)

## Data Availability

Not applicable.

## Supplementary Materials

The following are available online at https://www.mdpi.com/article/10.3390/plants10081703/s1, Figure S1: Geographical position of Golestan Province in Iran and distribution map of *Phalaris* genus, Circles represent *P. minor*, squares represent *P. brachystachys*, triangles represent *P. paradoxa*, Table S1: Geographical location, collection site of *Phalaris* genus biotypes
