# Supplementary material for: Point Mutations and Cytochrome P450 Can Contribute to Resistance to ACCase-Inhibiting Herbicides in Three Phalaris Species
Source: Plants (Basel). 2021 Aug 19;10(8):1703. doi: 10.3390/plants10081703 (PMC8401167; doi:10.3390/plants10081703)
Supplement: Supplementary file 1 [file plants-10-01703-s001.zip › plants-1326296-supplementary.pdf]

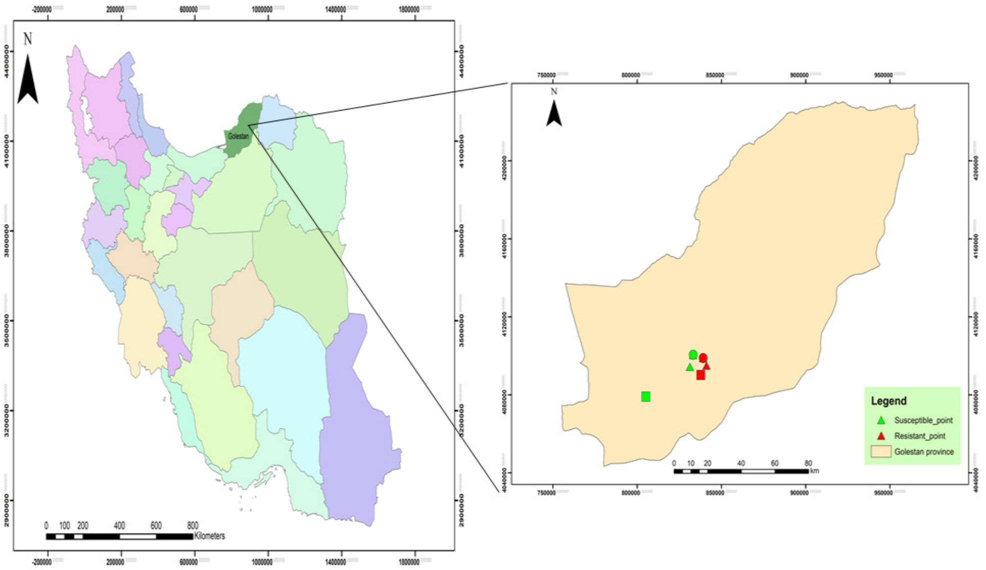

Figure S1. Geographical position of Golestan Province in Iran and distribution map of Phalaris genus, Circles represent *P. minor*, squares represent *P. brachystachys*, triangles represent *P. paradoxa*

Table S1. Geographical location, collection site of *Phalaris genus* biotypes

Species	Statues	Geographical location			Collection site
		Province	UTM coordinate		
P. minor	R	Golestan	304994	4094381	Wheat field
	S	Golestan	299195	4096390	Wheat field
P. paradoxa	R	Golestan	306742	4090452	Wheat field
	S	Golestan	296930	4090432	Wheat field
P. brachystachys	R	Golestan	303097	4085868	Wheat field
	S	Golestan	269860	4076834	Wheat field
